# Forces driving the three‐dimensional folding of eukaryotic genomes

**DOI:** 10.15252/msb.20188214

**Published:** 2018-06-01

**Authors:** Alvaro Rada‐Iglesias, Frank G Grosveld, Argyris Papantonis

**Affiliations:** ^1^ Center for Molecular Medicine Cologne University of Cologne Cologne Germany; ^2^ CECAD University of Cologne Cologne Germany; ^3^ Department of Cell Biology Erasmus Medical Center GE Rotterdam Netherlands

**Keywords:** chromatin, phase separation, RNA polymerase, TAD (topologically associating domain), transcription factor, Chromatin, Epigenetics, Genomics & Functional Genomics, Genome-Scale & Integrative Biology, Transcription

## Abstract

The last decade has radically renewed our understanding of higher order chromatin folding in the eukaryotic nucleus. As a result, most current models are in support of a mostly hierarchical and relatively stable folding of chromosomes dividing chromosomal territories into A‐ (active) and B‐ (inactive) compartments, which are then further partitioned into topologically associating domains (TADs), each of which is made up from multiple loops stabilized mainly by the CTCF and cohesin chromatin‐binding complexes. Nonetheless, the structure‐to‐function relationship of eukaryotic genomes is still not well understood. Here, we focus on recent work highlighting the biophysical and regulatory forces that contribute to the spatial organization of genomes, and we propose that the various conformations that chromatin assumes are not so much the result of a linear hierarchy, but rather of both converging and conflicting dynamic forces that act on it.

## Introduction

Chromatin in interphase nuclei is now understood to be spatially arranged in a multitude of loops (Dekker and Mirny, 2016; Knoch *et al*, 2016). However, the concept of chromatin looping is a rather old one: starting with spreads of “lampbrush” chromosomes from sperm (Gall & Murphy, 1998) and extending to interphase human cells (where the average “loop” length was estimated at ~ 86 kbp; Jackson *et al*, 1990).

In 2009, the development of Hi‐C, a whole‐genome variant of the chromosome conformation capture (3C) approach, allowed a reassessment of chromatin architecture at 1‐Mbp resolution (Lieberman‐Aiden *et al*, 2009). This revealed that chromosomal arms fold into alternating A‐ (mostly transcriptionally active) and B‐compartments (mostly inactive), which often preferentially interact with other compartments of the same type. This seminal study was followed by numerous others providing increasingly higher resolution views of spatial chromatin architecture. This way, sub‐Mbp domains harboring genomic segments that contact one another more frequently than segments in adjacent domains were uncovered and named “topologically associating domains” (TADs; Dixon *et al*, 2012; Nora *et al*, 2012; Sexton *et al*, 2012). Finally, even higher, sub‐kilobase, resolution Hi‐C experiments in human and mouse cells (Rao *et al*, 2014; Bonev *et al*, 2017) identified multiple “contact domains” at the sub‐TAD scale (185 kbp in size, on average). For approximately half of these contact domains, their boundaries almost exclusively showed strong looping between CTCF‐bound sites of convergent orientation (Rao *et al*, 2014). However, it should be noted that, in addition to the variable Hi‐C resolution in these studies, the different algorithms used to identify TADs (e.g., “directionality index” in Dixon *et al*, 2012) or contact domains (“arrowhead” in Rao *et al*, 2014) could also explain some of the reported divergence in domain size and boundary composition. Regardless, it is also important to consider that CTCF proteins only display dimerization potential in yeast‐two‐hybrid assays (Yusufzai *et al*, 2004), so additional partners would be required to stabilize such conformations. Thus, CTCF loops are stabilized by co‐bound cohesin complexes (Wendt *et al*, 2008; Rao *et al*, 2014) and facilitate interactions among genes and their cognate *cis*‐regulatory elements (Rao *et al*, 2014; Bonev *et al*, 2017). It is worth noting here that, although such “architectural” loops are often conserved between cell types (even between syntenic regions of different species), at the single‐cell level, they appear dynamic and highly variable (Hansen *et al*, 2017; Stevens *et al*, 2017). In addition, some CTCF loops are not constitutive, but rather cell type‐specific. This “dynamic” subset might involve several and non‐mutually exclusive mechanisms, such as: (i) tissue‐specific binding of CTCF mediated by cell type‐specific epigenetic modifications or transcription factors (Wang *et al*, 2012; Behera *et al*, 2018) and (ii) constitutively bound CTCF sites engaging into tissue‐specific interactions due to the action of additional “looping” co‐factors (Phillips‐Cremins *et al*, 2013; Huang *et al*, 2018).

On the basis of this new knowledge, whole‐genome conformation studies have been used to decipher how development or developmental disease (Dixon *et al*, 2015; Fraser *et al*, 2015; Lupiáñez *et al*, 2015; Franke *et al*, 2016; Bonev *et al*, 2017), cancer (Flavahan *et al*, 2016; Taberlay *et al*, 2016; Hnisz *et al*, 2017; Wu *et al*, 2017), DNA damage (Aymard *et al*, 2017; Canela *et al*, 2017), cellular aging (Criscione *et al*, 2016), and genetic variation (Javierre *et al*, 2016) impact on the structure and function of the genome. Needless to say that the advent of 3C technology (see overview in Denker & de Laat, 2016) has also provided insights into the higher order genomic organization of bacteria (e.g., Le & Laub, 2016; Lioy *et al*, 2018), fungi (e.g., Mizuguchi *et al*, 2014; Kim *et al*, 2017; Tanizawa *et al*, 2017), nematodes (e.g., Crane *et al*, 2015), the *Plasmodium falciparum* parasite (Ay *et al*, 2014), and plants (e.g., Dong *et al*, 2017). It is noteworthy that A‐/B‐compartments and TAD‐like structures can largely be identified across all organisms investigated to date.

Here, in light of recent data on perturbations of key architectural protein factors, on Hi‐C studies in single cells, and on computational modeling of 3D genome architecture, we surmise that a linear hierarchical model might not faithfully describe the complexity behind the multi‐layered architecture of eukaryotic genomes, and then discuss how chromatin identity and chromatin‐binding factors, transcriptional activity, and entropy may act as converging or opposing forces governing chromatin looping, phase separation, and functional genomic output.

## Topologically associating domains and their boundaries as “building blocks” of the genome

TADs were originally defined on the basis of 40‐kbp resolution Hi‐C maps, and this showed an average of ~ 3,000 such insulated domains in each of the various mammalian cell types tested (Dixon *et al*, 2012, 2015). The key functional attribute that follows the existence of TADs is that they facilitate spatial interactions of sequences within the domain, namely between gene promoters and cognate enhancers, while simultaneously insulating those sequences from spurious interactions with genomic segments outside the TAD (Symmons *et al*, 2014, 2016). Nonetheless, due to the fact that TAD detection is sensitive to the combination of the data resolution and algorithm used, their biological significance and robustness has been debated. However, four independent lines of evidence have emerged that validate TADs as true functional entities. First, multi‐scale computational interrogation of TADs and their insulation potential in Hi‐C data showed that they represent a distinct functionally privileged scale of organization arising from their ability to partition interactions (Zhan *et al*, 2017). Second, a crosslinking‐free 3C approach, i3C, showed that the topological restrictions imposed by TADs hold true natively in mammalian nuclei (Brant *et al*, 2016). Third, the deterioration or duplication of TAD boundaries *in vivo* led to obvious ectopic interactions and gene misexpression (Lupiáñez *et al*, 2015; Franke *et al*, 2016; Hnisz *et al*, 2016; Narendra *et al*, 2016). Fourth, insulation at TAD boundaries is markedly affected upon loss of structural proteins previously proposed to control their establishment (i.e., CTCF, cohesin; Zuin *et al*, 2014; Haarhuis *et al*, 2017; Nora *et al*, 2017; Rao *et al*, 2017; Schwarzer *et al*, 2017).

Overall, the most striking finding with respect to TADs has been their apparent robustness when comparing boundary positions between species and/or conditions. For instance, upon stem cell differentiation (Dixon *et al*, 2015; Fraser *et al*, 2015), reprogramming (Beagan *et al*, 2016; Krijger *et al*, 2016), or cytokine stimulation (Jin *et al*, 2013; Le Dily *et al*, 2014), TADs exhibit only limited changes (e.g., ~ 11% shifted at least one boundary upon treatment with TNFα). Nevertheless, this limited variation in TAD boundaries can be associated with expression changes in key cell identity genes (Bonev *et al*, 2017; Stadhouders *et al*, 2018) and in “compartment switching” (Fraser *et al*, 2015) illustrating again the importance of TAD‐imposed topological restrictions in gene expression control. Similarly, comparative analysis of Hi‐C data from four mammals revealed that the partitioning of chromosomes into TADs is conserved once syntenic regions are considered. This conservation coincides with the presence of conserved CTCF/cohesin‐binding sites (Vietri Rudan *et al*, 2015), which is remarkable considering how transcription factors typically display species‐specific binding even when the underlying sequences are conserved (Schmidt *et al*, 2010) and probably indicates the functional relevance of these CTCF/cohesin‐bound sites. Given that evolutionary divergence of higher order structure in vertebrates is associated with changes in the well‐insulated TADs harboring developmental loci (Chambers *et al*, 2013; Acemel *et al*, 2016; Guerreiro *et al*, 2016), the composition of TAD boundaries becomes a critical component. This begs the question: What stabilizes TADs as “meta‐stable” formations, and how is their insulator potential realized?

Initially, ~ 10% of TADs identified in mammals were bound by CTCF (see Dixon *et al*, 2012, 2015). Hi‐C studies of increasingly higher resolution, also coupled to targeted degradation of CTCF, have revealed a larger fraction of TAD boundaries bound and/or reliant on CTCF (Rao *et al*, 2014; Nora *et al*, 2017). Nonetheless, a comparable number of TAD boundaries appear to be CTCF‐independent and are instead demarcated by active RNA polymerases, transcriptional activators, nascent RNA, and/or by transitions in chromatin states/compartments (Dixon *et al*, 2012; Rao *et al*, 2014; Bailey *et al*, 2015; Bonev *et al*, 2017; Nora *et al*, 2017). In addition, boundaries where CTCF co‐associates with bound topoisomerase II (Uusküla‐Reimand *et al*, 2016), RUNX1 (Barutcu *et al*, 2016), BRD2 (Hsu *et al*, 2017), YY1 (Beagan *et al*, 2017; Weintraub *et al*, 2017), or the nuclear matrix protein HNRNPU (Fan *et al*, 2017) have now also been uncovered. Along the same lines, TAD boundaries in the fruit fly are less likely to be marked by insulators like dCTCF, BEAF, and Su(Hw), and more likely to harbor constitutively active loci (Ulianov *et al*, 2016; Rowley *et al*, 2017) and to coincide with transitions between A‐ and B‐compartments (Rowley *et al*, 2017). TAD formation coincides spatially and temporally with transcriptional activation of the genome either following heat shock recovery (Li *et al*, 2015) or zygotic genome activation (Hug *et al*, 2017) in fruitflies. In fact, use of transcriptional inhibitors in Drosophila embryos results in loss of insulation at TAD boundaries commensurate with the loss of bound RNA polymerase; assuming inhibitors work efficiently, engagement of a gene TSS with the polymerase (and not transcriptional elongation) suffices to already confer a “boundary‐like” insulation effect (Hug *et al*, 2017). Equally, domain boundaries might not directly involve bound RNA polymerase or transcriptional activity, but rather arise due to spatial segregation between active and inactive chromatin compartments (Rowley *et al*, 2017). Nevertheless, the close relationship between transcription and chromatin architecture is well illustrated in studies where transcriptional data sufficed for correctly predicting 3D genome folding (Rowley *et al*, 2017; Rennie *et al*, 2018).

Along the same lines, numerous organisms lack orthologues of CTCF, but do exhibit insulated TAD‐like domains in Hi‐C experiments; these include *Caenorhabditis elegans* (Crane *et al*, 2015), *Arabidopsis thaliana* (Dong *et al*, 2017), *Schizosaccharomyces pombe* (Mizuguchi *et al*, 2014), or *Caulobacter crescentus* and *Escherichia coli* (Le & Laub, 2016; Lioy *et al*, 2018). For example, in an elegant genome editing experiment, insertion of the strongly expressed *rsaA* bacterial gene in the middle of another TAD gave rise to a novel boundary, the strength of which was progressively diminished as the transcribed sequence of the inserted gene was shortened (Le & Laub, 2016). Nonetheless, examples have now also been described of (predominantly developmental) loci where transcriptional engagement does not suffice to generate a boundary (Bonev *et al*, 2017), or where changes in insulation precede transcriptional changes but are concomitant with chromatin state remodeling (Stadhouders *et al*, 2018). Finally, a notable exception to the above is the transcriptionally inert mammalian zygote, where TADs and “architectural” loops, but not compartments, could be detected in maternal chromatin (Flyamer *et al*, 2017) and perhaps emerge concomitantly with DNA replication (Ke *et al*, 2017). This last remark is in line with a single‐cell Hi‐C study of the cell cycle in haploid ES cells. Therein, chromatin loops appear relatively stable throughout the cell cycle, but TAD boundaries weaken after the G1 phase, and give way to increasing compartmentalization that peaks just before mitosis (Nagano *et al*, 2017).

Taken together, although TADs seem to represent universal blocks of genome organization, the preponderant mechanisms involved in their establishment and maintenance seem to have gained in complexity during evolution, with transcriptional activity, inactivity, and the associated chromatin/compartment states being decisive contributors to genomic partitioning. Nonetheless, the different insulation mechanisms found in different organisms (e.g., the presence of multiple *bona fide* insulators in Drosophila compared to the pervasiveness of CTCF in mammals; Rowley *et al*, 2017) might be related to changes in genome size. Smaller genomes, in which genes and their cognate regulatory elements tend to be closer together (like the fruit fly), display topological domains determined by transcriptional/chromatin state and local, short‐range, insulation; larger genomes (e.g., mouse, human), in which regulatory elements may be positioned Mbp away from their target genes, contain CTCF‐dependent boundaries that enable mixing of loci displaying different chromatin/compartment states and thus facilitate the establishment of long‐range interactions while insulating them from flanking TADs (Symmons *et al*, 2016; Nora *et al*, 2017; Rowley *et al*, 2017).

## CTCF and cohesin in insulation, loop formation, and long‐range contact facilitation

High‐resolution Hi‐C analyses in mammals have decisively related the presence of CTCF and cohesin complexes at the bases of chromatin loops both at TAD boundaries and within TADs themselves (e.g., Rao *et al*, 2014; Bonev *et al*, 2017). First, precision genome editing studies showed that looping does require convergently positioned pairs of CTCF‐bound sites (Guo *et al*, 2015; de Wit *et al*, 2015). Then, systems for auxin‐mediated protein degradation were employed to acutely and reversibly deplete CTCF and cohesin, thus enabling an evaluation of their regulatory role and their direct impact on 3D chromatin organization while avoiding problems associated with either their partial depletion (e.g., siRNA) or constitutive loss (i.e., secondary effects).

The rapid degradation of CTCF in (dividing) mouse ES cells and in (non‐dividing) astrocytes led to a gradient of insulation loss at ~ 80% of TAD boundaries (with the highly insulated developmental loci being primarily affected), as well as to a loss of looping between CTCF/cohesin sites (Nora *et al*, 2017). In a parallel study of the same mESC system, far less dramatic effects on TAD insulation were observed (albeit with somewhat lower CTCF degradation efficiency; Preprint: Kubo *et al*, 2017). Nonetheless, effects in either study were fully reversible once degradation was attenuated, and A/B‐compartmentalization was only mildly affected in either cell type. This is indicative of TAD formation and compartmentalization representing two independent mechanisms of chromatin folding, with only the former being CTCF‐dependent in mammals. The loss of CTCF and, consequently of many insulation boundaries, did not have an immediate impact on gene expression (370 genes were differentially expressed after 1 day of auxin treatment). Although the larger gene expression changes observed at later CTCF depletion times could arise due to secondary effects, some of them could still be directly mediated by CTCF but required more time to fully manifest. Immediate gene downregulation was mostly observed at genes having CTCF bound at promoter‐proximal regions that were not typically found at TAD boundaries. In contrast, immediate gene upregulation was mainly observed for genes that CTCF was predicted to insulate from enhancers located in neighboring TADs. Therefore, as regards TADs, CTCF function seems to be particularly important for insulation from spurious gene–enhancer interactions, while its role in the maintenance of gene expression (e.g., in facilitating long‐range gene–enhancer interactions) does not appear to be equally critical. Still, deleting the CTCF‐dense and evolutionarily conserved TAD boundary at the *Firre* locus did not alter local insulation, in contrast to deletion of *Firre* itself, suggesting that additional mechanisms may act to confer insulation between consecutive TADs (Barutcu *et al*, 2018).

**Figure 1:**
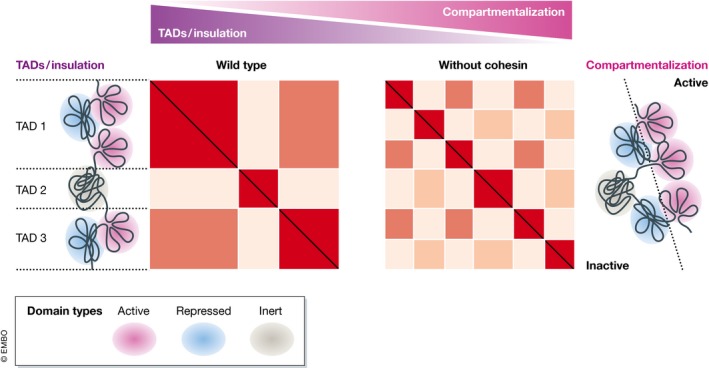
Insulated topological domains versus compartments Typical Hi‐C maps (“wild type”) reveal alternating topological domains (TADs; *red*) that insulate the chromatin domains each TAD contains from domains in neighboring TADs. Once members of the cohesin complex are depleted from cells, Hi‐C maps (“without cohesin”) are dominated by contacts between compartments that exhibit the propensity to interact with one another on the basis of their transcriptionally active or repressed/inert identity.

Similar auxin‐mediated degradation or conditional genetic deletions of different subunits of the cohesin complex (SMC1A, WAPL, NIPBL, or PDS5) *in vivo* and *in vitro* were also performed recently. All converged to the same result: Quantitative elimination of nearly all DNA‐bound cohesin complexes leads to the loss of essentially all “contact domains” that relied on CTCF and cohesin (Haarhuis *et al*, 2017; Rao *et al*, 2017; Schwarzer *et al*, 2017; Wutz *et al*, 2017). However, not all chromatin contacts were eliminated: Interactions reminiscent of A‐/B‐compartmentalization were strongly accentuated (Fig 1). Moreover, the acute loss of “contact domains” stabilized by CTCF and cohesin had minor (within 6 h of auxin treatment) effects on gene expression (e.g., < 70 genes changed more than twofold in cells where all SMC1A was degraded; Rao *et al*, 2017). Upon permanent loss of the cohesin complex (via *Nipbl* deletion in mouse liver), gene expression changes were more profound (~ 1,000 genes), but still moderate considering the overall effect on TAD organization. Both up‐ and downregulation of genes were observed, mostly as a result of ectopic enhancer–gene interactions and loss of long‐range gene–enhancer communication, respectively. Nevertheless, as the majority of liver genes did not change their expression levels even after this prolonged loss of cohesin, additional mechanisms might be invoked to ensure gene expression homeostasis. For example, enhancer “hijacking” effects might be minimized by gene–enhancer incompatibilities, by compartmentalization (i.e., by spatial segregation of loci of different chromatin states, like inactive promoters contacting active enhancers), or by CTCF‐dependent but cohesin‐independent insulation. Similarly, although cohesin and TADs may facilitate the establishment of long‐range enhancer–gene interactions, these might still occur at most genes on the basis of additional non‐mutually exclusive mechanisms (e.g., via YY1 or compartmentalization), thus enabling gene expression homeostasis and interaction specificity. One possibility is that the topological restrictions imposed by cohesin and TADs might be particularly important in the induction, rather than in the maintenance, of gene expression—i.e., they may mostly act to facilitate “first‐time” promoter–enhancer encounters.

As the increased compartmentalization observed upon cohesin loss is not seen once CTCF is depleted, one can deduce that the cohesin complex might be sufficient to counteract excessive compartmentalization and thus, dictate communication between loci. Upon knockout of the WAPL cohesin‐release factor for example, enlargement of chromatin loops is strongly affected (Haarhuis *et al*, 2017). This, and similar results, begs the question: How might the contribution of cohesin to interphase chromatin folding be explained and reconciled with dynamic loop length? The currently most discussed model is that of “loop extrusion”, which had been previously proposed (Nasmyth, 2011) and has now been repurposed to apply to the formation of “contact domains” (Fudenberg *et al*, 2016; Gassler *et al*, 2017). Although it remains unclear whether one or two cohesin rings are required to bring together two chromatin segments, the idea is that loops enlarge as the cohesin complex progress along the fiber (until it meets a physical barrier, which in many cases will involve CTCFs bound on either fiber). Hence, loop extrusion would require either a motor on cohesin itself or on some other chromatin‐bound factor. Cohesin does possess an ATPase domain (Nasmyth, 2011), which may be able to take up this role (just like the yeast condensin motor that extrudes DNA asymmetrically and is therefore incompatible with current loop extrusion models; Ganji *et al*, 2018). Another possibility would be the most processive RNA polymerase, which in the ~ 22 min that cohesin remains bound to DNA could extrude loops up to 875 kbp in length (assuming an average speed of 3.5 kbp/min; Wada *et al*, 2009). This possibility would also be in agreement with cohesin positioning being regulated by the very act of transcription (Busslinger *et al*, 2017) and its ensuing supercoiling (Racko *et al*, 2018), as well as by the increased mobility of genes and enhancers once they transition from an inactive to an active state (Gu *et al*, 2018). More recently, a model was proposed whereby simple diffusion, biased by multiple cohesin loading events at the same loading site, might be sufficient for loop extrusion and its prolongation (to explain Mbp‐long loops; Brackley *et al*, 2018). This diffusion model can, at least in part, address the question of how cohesin also accumulates at the boundaries of TADs harboring inactive genes (e.g., Polycomb targets).

Still, despite all these new insights on CTCF/cohesin‐mediated loops in mammalian genomes, the question remaining open is as follows: Which are the driving forces behind the extensive and complex patterns of contacts revealed once cohesin and/or CTCF are depleted from cell nuclei? For example, most active genes, including cell type‐specific ones, do not seem to be severely affected by either CTCF or cohesin depletion, indicating that gene–enhancer interactions overall persist. Similarly, transcriptionally inert parts of the genome, in both facultative and constitutive heterochromatin, remain largely inactive and do not spread into active ones upon loss of CTCF or cohesin. Interestingly, both CTCF and cohesin display long residence times of on DNA (~ 2 and ~ 22 min, respectively) and different search times when unbound (CTCF rapidly rebinds within ~ 1 min, while cohesin requires > 30 min; Hansen *et al*, 2017), which hints toward divergent dynamics impinging on chromatin folding. Hence, we discuss below some of the mechanisms that contribute to chromatin topology independently of (or in parallel to) CTCF/cohesin.

## Heterochromatinization as a compartmentalization force

Facultative heterochromatin spreads along a substantial part of eukaryotic chromosomes, and Polycomb group (PcG) proteins form multiprotein complexes (on the basis of PRC1 and PRC2) that play essential roles during development due to their capacity to modify chromatin and repress gene expression. Critically, PcG complexes form nuclear compartments that vary in size and number and have been referred to as PcG bodies or foci (Cheutin & Cavalli, 2012; Wani *et al*, 2016). Most recently, 3C‐based studies revealed that these PcG bodies most likely represent PcG‐bound loci interacting both in *cis* and in *trans*, and residing in spatial proximity (Denholtz *et al*, 2013; Joshi *et al*, 2015; Schoenfelder *et al*, 2015; Cruz‐Molina *et al*, 2017; Eagen *et al*, 2017; Kundu *et al*, 2017). Also, “chromosomal walks” permitting identification of multi‐way (and not just pairwise) contacts showed that the Polycomb‐bound *Hox* domains fold into larger hubs (Olivares‐Chauvet *et al*, 2016). These highlight that PcG complexes are strong mediators of chromatin interactions and, thus, of nuclear architecture. Interestingly, the folding and compacting properties of Polycomb‐bound chromatin appear to be unique compared to other chromatin compartments, but also variable between cell types. For example, Drosophila Polycomb domains exhibit the densest packing, which also increases commensurate to the length of the corresponding domain. They strongly segregate from their neighboring active domains and display disparate self‐organization properties to both euchromatin and constitutive heterochromatin (Boettiger *et al*, 2016). In mammals, Polycomb‐bound chromatin segregates into specific sub‐compartment and typically occupies central nuclear positions (i.e., does not associate with the lamina), again supporting distinct folding principles compared to active and constitutively inactive chromatin (Rao *et al*, 2014; Vieux‐Rochas *et al*, 2015). Furthermore, during mouse ESC differentiation into cortical neurons, Polycomb‐bound chromatin switches from the A‐ to the B‐compartment—which is accompanied by a strong loss of PRC1 binding and *cis*‐ and *trans*‐interactions between Polycomb‐bound loci (Bonev *et al*, 2017) and suggests a transition to a constitutive heterochromatic state. Congruently, poised enhancers and bivalent genes marked by H3K27me3 in ESCs establish local spatial interactions that confer a permissive regulatory topology to certain developmental loci to ensure ensuing activation upon differentiation (Cruz‐Molina *et al*, 2017). Overall, long‐range interactions between Polycomb‐bound loci appear more prevalent in mouse stem cells and undifferentiated progenitors than in non‐dividing and fully differentiated cells (Joshi *et al*, 2015; Schoenfelder *et al*, 2015; Vieux‐Rochas *et al*, 2015). These long‐range interactions might act to repress developmental genes, while keeping them in accessible nuclear compartments (i.e., central nuclear locations), permissive for activation upon appropriate cues (Vieux‐Rochas *et al*, 2015). Thus, the structural and functional properties of Polycomb‐bound chromatin might differ among species and cell types.

**Figure 2:**
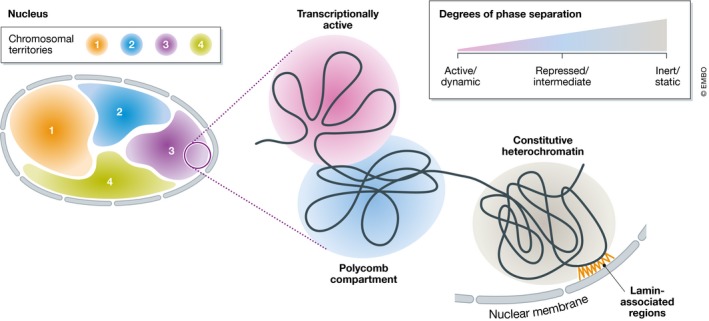
Chromatin identities and phase separation shape the 3D genome Chromosomes occupy distinct territories in the cell nucleus (*left*), and each such territory is partitioned into sub‐Mbp domains. Transcriptionally active ones are most dynamic and are brought about by the interplay of chromatin with RNA polymerases, transcription factors (e.g., YY1 or AP‐1), and chromatin‐modifying enzymes (e.g., Trithorax). Transcriptionally inert loci in constitutive heterochromatin are the least dynamic and most strongly phase‐separated, and arise via interactions with the lamina and with heterochromatic factors (e.g., HP1α). Repressed loci form “Polycomb bodies/compartments”, which display intermediate dynamics and form on the basis of interactions with the PRC1/2 complexes.

Upon heat shock, Drosophila nuclei undergo a dramatic reorganization at both the TAD and intra‐TAD levels. Numerous deactivated genes form strong interactions with other deactivated genes and enhancer elements now marked by Polycomb (Li *et al*, 2015). This suggests that this “spatial decommissioning” is not only a driving force of genomic reorganization, but also one that acts to preserve potentially functional enhancer–gene interactions in anticipation of an ensuing reactivation signal. So, what are the molecular mechanisms that enable PcG‐bound chromatin to acquire its unique and apparently relevant topological features? First of all, both *cis‐* and *trans*‐interactions between PcG‐bound loci seem to be fully dependent on the presence of intact PRC1 and PRC2 complexes (Joshi *et al*, 2015; Schoenfelder *et al*, 2015; Cruz‐Molina *et al*, 2017). PRC1 might be the main mediator of these interactions (Schoenfelder *et al*, 2015; Bonev *et al*, 2017), while PRC2 might be required to efficiently recruit PRC1 to its target regions (Joshi *et al*, 2015). More specifically, once recruited to its genomic targets, the PRC1 “polyhomeotic” subunits can mediate both local and long‐range interactions between PcG‐bound loci due to multimerization via their sterile alpha motif (SAM) domains (Isono *et al*, 2013; Wani *et al*, 2016). Additional multivalent interactions between PRC1 and PRC2 subunits, as well as with nearby nucleosomes, help compact chromatin and further stabilize the associations between PcG‐bound loci (Grau *et al*, 2011; Blackledge *et al*, 2014), ultimately resulting in the formation of discrete nuclear PcG bodies (Fig 2). Interestingly, the repressive identity of H3K27me3‐marked genomic regions can be robustly reversed by Polycomb clearance initiated by and dependent on distal enhancers (Saxena *et al*, 2018), probably due to the action of H3K27me3 demethylases, as well as the inhibitory role of nascent RNA on PcG binding and/or activity (Beltran *et al*, 2016). This shows how the two states, active and Polycomb‐inactive, dynamically compete for nucleating active or inactive micro‐compartments that spatially segregate chromatin in eukaryotic nuclei.

Along the same lines, constitutive heterochromatin produces spatially distinct “phase‐separated” micro‐compartments in mammalian nuclei, where inactive sequences cluster (Larson *et al*, 2017; Strom *et al*, 2017). The structural basis for this is the bivalency of HP1α that allows this protein to simultaneously interact with two nucleosomes, which together with the dimerization capacity of HP1α results in a high local concentration of HP1α proteins clustering heterochromatic regions together (Fig 2). In light of the capacity of PcG complexes to polymerize and establish multivalent interactions (including some mediated by low sequence complexity domains), it is then tempting to speculate that PcG bodies might also represent micro‐compartments formed by phase separation (Isono *et al*, 2013; Wani *et al*, 2016). However, there are some clear differences between constitutive heterochromatin (marked by HP1α and H3K9me3) and PcG compartments: Polycomb‐bound loci can form clusters involving frequent and robust *trans*‐interactions (Joshi *et al*, 2015; Schoenfelder *et al*, 2015; Vieux‐Rochas *et al*, 2015; Bonev *et al*, 2017), while constitutively heterochromatic loci display considerably less spatial clustering and long‐range interactions (Beagrie *et al*, 2017; Stevens *et al*, 2017). This is also in line with their frequent association with the lamina at the nuclear periphery. The overall differences between Polycomb‐bound and constitutive heterochromatin are well illustrated by experiments where tethering of EZH2‐ or SUV39H1‐binding platforms within an active TAD leads to the establishment of new spatial contacts only with other EZH2‐ or SUVE39H1‐bound regions, respectively (Wijchers *et al*, 2016).

## Transcription as a looping force

Does transcriptional activity also drive looping and spatial clustering of genomic loci like Polycomb or HP1α proteins and would this be through either phase separation (Hnisz *et al*, 2017) or some analogous mechanism? The majority of 3C‐based studies performed to date are in support of widespread interactions among gene promoters and *cis*‐regulatory elements. For example, in contact maps from numerous primary human tissues and cell types, a subset of regions rich in strong enhancer clusters (“LCRs/super‐enhancers”) and active genes were seen interacting unusually frequently. These co‐interacting regions, called “FIREs”, display tissue specificity, only partially involve CTCF and cohesin binding, and are thus central to the conformation of the active compartment in the different cell types (Schmitt *et al*, 2016; Thibodeau *et al*, 2017). This finding was confirmed by an orthogonal, ligation‐free, approach called GAM (“genome architecture mapping”; Beagrie *et al*, 2017). In GAM data from mouse ES cells, which also permits for multi‐way contacts to be identified, the most prominent contacts involved super‐enhancers and active genes. The formation of such clusters has also been observed for sequences bound by pluripotency transcription factors (both in *cis* and in *trans*; de Wit *et al*, 2013) or carrying differentially activated tRNA genes during macrophage differentiation (van Bortle *et al*, 2017). Such transcription “hubs” or “factories” are known nucleoplasmic entities, with a ~ 1,000‐fold local increase in RNA polymerase concentration, that remain stable over hours (Kimura *et al*, 2002; Ghamari *et al*, 2013), and harbor numerous loops around them (see Papantonis & Cook, 2013 for a review). For instance, ChIA‐PET experiments focusing on contacts made by active RNA polymerase II have unveiled an emerging theme in 3D genomic architecture: preferential spatial associations between co‐transcribed and co‐regulated genes in response to signaling (Li *et al*, 2012; Papantonis *et al*, 2012). Similarly, a subset of promoter–promoter interactions exerting unidirectional regulatory activity on one another (Dao *et al*, 2017), and enhancers that follow transcribing RNA polymerases along gene bodies to form dynamic spatial configurations (Larkin *et al*, 2012; Lee *et al*, 2015). Thus, it is tempting to speculate that chromatin compartments with different transcriptional activity might display different and tunable degrees of “phase separation” (Hnisz *et al*, 2017), depending on the type, strength, and dynamics of the physical interactions in each compartment (Fig 2). However, biophysical evidence supporting liquid phase separation of transcriptionally active domains remains sparse (Preprint: Hilbert *et al*, 2017) and their formation might not necessarily involve such separation.

The formation of such phase‐separated transcriptionally active compartments on the basis of chromatin interactions and high local concentrations of RNA, transcription factors, and the relevant machinery is exemplified by the nucleolus. In computational models, spatial associations among repetitive rDNA loci aided by multimeric binding of UBF suffice to give rise to a single nucleolar compartment—and this model was experimentally validated in yeast (Grob *et al*, 2014; Hult *et al*, 2017). At a smaller scale, the formation of “histone locus bodies” in the fruit fly (Salzler *et al*, 2013) or histone gene factories in human cells (Li *et al*, 2012) occurs (and also ectopically) only when they are transcriptionally active and insulated from surrounding domains. Recently, enhancer–promoter interactions and higher order compartmentalization, driven by non‐coding transcription in T cells or by BAF complexes interacting with EWS‐FLI1 in cancer cells, were proposed to instruct phase transition and organize chromatin (Boulay *et al*, 2017), albeit that this was not formally demonstrated. Also, small chromosomal segments spatially cluster with other segments of the same chromatin class (van de Werken *et al*, 2017), and transcriptional initiation at a tagged locus resulted in its spatial confinement, maintained even when transcriptional elongation was inhibited (Germier *et al*, 2017). Thus, in addition to modeling of the non‐specific, entropy‐based, “depletion attraction” mechanism that can explain clustering of DNA‐bound RNA polymerases and transcription factors to stabilize loops (Marenduzzo *et al*, 2006), these data advocate in favor of an assembly of transcriptional “hubs” or “factories” where loops among active genes and regulatory elements are located. We propose that this applies across active loci and explains fine‐scale A‐compartments and enhancer–gene communication both within and across TADs. Evidence supporting MLL/Trithorax complexes, associated histone modifications, and transcription factors as mediators of such phase transition is now emerging (Bonev *et al*, 2017; Weintraub *et al*, 2017; Yan *et al,* 2018; Huang *et al*, 2018; Rennie *et al*, 2018). Importantly, rather than being necessary for the establishment of these active compartments and the interactions therein, cohesin might either play a secondary role or even act to oppose them (Rao *et al*, 2017; Schwarzer *et al*, 2017).

The correlation between transcription and loop formation had already been evidenced by supercoils disappearing upon maturation of erythroblasts into transcriptionally inert erythrocytes (Cook & Brazell, 1976). In Drosophila, chromosomal segments recovering from heat shock nucleate the 3D organization of the whole genome upon transcriptional activation, and the transcriptional state of “compartmental domains” faithfully predicts steady‐state contacts seen by Hi‐C (Li *et al*, 2015; Rowley *et al*, 2017). At the same time, obvious correlation between co‐expression domains or transcriptionally active repetitive elements and spatial genome organization has also been documented across mammalian cell types (Cournac *et al*, 2016; Belyaeva *et al*, 2017; Soler‐Oliva *et al*, 2017), while interactions between TADs (“meta‐TADs”) do align with the switch in gene expression programs between mouse ES cells and neurons (Fraser *et al*, 2015).

However, in the numerous examples of transcription factor‐stabilized looping emerging, not all loops are dynamically changing in response to extracellular cues. Interactions involving gene promoters and cognate enhancers can either be pre‐established (“pre‐looped”; Ghavi‐Helm *et al*, 2014; Cruz‐Molina *et al*, 2017), fully static, or dynamic in response to TNFα signaling (mediated by NF‐κB; Kolovos *et al*, 2016), during macrophage development (mediated by AP‐1; Phanstiel *et al*, 2017), differentiation of the epidermis (mediated by ETS family factors; Rubin *et al*, 2017), upon adipogenesis (via both activators like C/EBP and co‐repressors like NuRD; Siersbæk *et al*, 2017), or by the homotypic interplay of poised and active loops in the mouse *HoxB* locus (Barbieri *et al*, 2017). The prevalence of pre‐looped versus *de novo* enhancer–gene interactions during gene induction might be locus‐ and cell type‐dependent, with either permissive or instructive regulatory principles dominating, respectively. In this regard, the timely and robust first‐time encounters between genes and their cognate enhancers can be facilitated by either pre‐formed contacts (Ghavi‐Helm *et al*, 2014; Kolovos *et al*, 2016; Cruz‐Molina *et al*, 2017) or increased mobility due to the activity of the polymerase and/or cohesin (Busslinger *et al*, 2017; Gu *et al*, 2018). Finally, along a time course of B‐cell reprogramming, it is transcription factors like Nanog that instruct loop and TAD reorganization, often before changes in gene expression (Stadhouders *et al*, 2018), but always concomitant with chromatin remodeling and increased accessibility, which was shown to suffice for altering nuclear organization (Therizols *et al*, 2014). In all the above cases, CTCF occupancy and CTCF‐based loops fail to fully predict chromatin folding and its ensuing dynamics, while the key predictor of architectural changes at multiple levels is the engagement of genomic loci with transcription factors, chromatin remodelers, and/or the RNA polymerase (even in its “poised” state; see Fig 2). Still, for many of the aforementioned factors, conclusive evidence demonstrating their instructive role in mediating long‐range interactions is still missing; gain‐of‐function approaches based on tethering of candidate proteins (e.g., using dCas9‐fusions) to specific loci, as well as genetic deletion of specific transcription factor‐binding sites, might help in this respect.

Finally, insights on how these spatial interactions might give rise to the contact maps seen by Hi‐C come from simulations of chromatin folding that allow individual conformations to form. This is important because chromatin folding in interphase nuclei seems to be highly heterogeneous (as seen by single‐cell Hi‐C studies; Flyamer *et al*, 2017; Nagano *et al*, 2017), while “ChromEMT” imaging of native chromatin revealed a largely unstructured ~ 10‐nm fiber that forms loops and chromatin clusters differentially across the cell population (Ou *et al*, 2017). Thus, even without invoking “loop extrusion”, binding of proteins (simulated by spheres) to the chromatin fiber (simulated by a string of beads) gives rise to loops. Strikingly, chromatin‐bound factors of the same “identity” will cluster spontaneously to form multi‐loop structures that explain much of the structure seen in TADs and/or A‐/B‐compartments (Barbieri *et al*, 2013; Brackley *et al*, 2016, 2017). Of course, this requires both multivalency and “on/off” binding cycles from the protein factor with a propensity to rebind the same cluster (as seen for Sox2 by live cell imaging; Liu *et al*, 2014), and it appears that much of the information required for the proper spatial folding of chromosomes is encoded in the epigenetic profiles marking their active and inactive stretches (Di Pierro *et al*, 2017), and even pure mechanical forces can profoundly impact both epigenetic and higher order characteristics of chromosomes (Le *et al*, 2016; Stephens *et al*, 2018).

## Conclusions and outlook

The advances that the development of 3C technology, super‐resolution imaging, and sophisticated *in silico* modeling has brought about in the last decade now allow us to revisit essentially every aspect of nuclear organization and function. Of course, and despite these advances, a series of question remain unaddressed or need to be reassessed under new light. To name a few: Can we identify new factors (or new roles for known factors) that contribute to the diverse repertoire of 3D genome folding? How does insulation occur at boundaries between transcriptionally inactive TADs? Is there a need for “bookmarking” of topological elements so that they may serve as nucleating points for the re‐emergence of 3D chromatin folding after exit from mitosis? How would cells lacking important architectural proteins, like CTCF and cohesin, respond if not tested under “steady‐state” conditions, but rather if forced to execute a switch in their gene expression program? What are the different biophysical characteristics of the various micro‐environments that comprise the interphase nucleus, and how do these simultaneously allow for robust yet heterogeneous transcriptional profiles in a cell population? Working toward addressing such questions will allow us to further dissect a fundamental question of modern biology: How the spatio‐temporal organization of chromosomes has evolved to accommodate the functional needs of the different eukaryotic cell types.

## Conflict of interests

The authors declare that they have no conflict of interest.
